# Dew benefits on alpine grasslands are cancelled out by combined heatwave and drought stress

**DOI:** 10.3389/fpls.2023.1136037

**Published:** 2023-05-09

**Authors:** Yafei Li, Werner Eugster, Andreas Riedl, Marco M. Lehmann, Franziska Aemisegger, Nina Buchmann

**Affiliations:** ^1^ Institute of Agricultural Sciences, ETH Zurich, Zurich, Switzerland; ^2^ Forest Dynamics, Swiss Federal Institute for Forest, Snow and Landscape Research (WSL), Birmensdorf, Switzerland; ^3^ Institute for Atmospheric and Climate Science, ETH Zurich, Zurich, Switzerland

**Keywords:** heatwave, dew, alpine grassland, drought, stable isotope, net ecosystem production

## Abstract

Increasing frequencies of heatwaves combined with simultaneous drought stress in Europe threaten the ecosystem water and carbon budgets of alpine grasslands. Dew as an additional water source can promote ecosystem carbon assimilation. It is known that grassland ecosystems keep high evapotranspiration as long as soil water is available. However, it is rarely being investigated whether dew can mitigate the impact of such extreme climatic events on grassland ecosystem carbon and water exchange. Here we use stable isotopes in meteoric waters and leaf sugars, eddy covariance fluxes for H_2_O vapor and CO_2_, in combination with meteorological and plant physiological measurements, to investigate the combined effect of dew and heat-drought stress on plant water status and net ecosystem production (NEP) in an alpine grassland (2000 m elevation) during the June 2019 European heatwave. Before the heatwave, enhanced NEP in the early morning hours can be attributed to leaf wetting by dew. However, dew benefits on NEP were cancelled out by the heatwave, due to the minor contribution of dew in leaf water. Heat-induced reduction in NEP was intensified by the combined effect of drought stress. The recovery of NEP after the peak of the heatwave could be linked to the refilling of plant tissues during nighttime. Among-genera differences of plant water status affected by dew and heat-drought stress can be attributed to differences in their foliar dew water uptake, and their reliance on soil moisture or the impact of the atmospheric evaporative demand. Our results indicate that dew influence on alpine grassland ecosystems varies according to the environmental stress and plant physiology.

## 1 Introduction

A record-breaking heatwave struck Europe in June 2019 (Mitchell et al., 2019; WMO, 2019), one in a series of severe heatwaves and droughts since summer 2003 (Ciais et al., 2005), 2010 (Barriopedro et al., 2011), 2016 (Zschenderlein et al., 2018), and 2018 (Gharun et al., 2020). Drought often is associated with a concurrent heatwave that affects terrestrial ecosystems (Overpeck, 2013), creating a so-called compound extreme event or period (Zscheischler and Fischer, 2020; Zscheischler et al., 2020). Compared to forests, grasslands are less vulnerable to drought stress, because of their relatively stable water use efficiency (i.e., the ratio of gross primary productivity per unit ecosystem evapotranspiration; Wolf et al., 2013) during a drought period. However intense and prolonged droughts and heatwaves do negatively affect grasslands (e.g., Gharun et al., 2020). A heatwave increases evapotranspiration of grasslands, thereby relieving the vegetation from heat stress, but at the expense of available water supply, which becomes scarcer the longer the heatwave persists (Teuling et al., 2010). Cremonese et al. (2017) reported that the combined drought and heat stress caused a reduction of canopy greenness in a mountain grassland. Also, De Boeck et al. (2016) reported that a heatwave combined with drought stress caused a reduction in above-ground biomass of alpine grassland plants. Li et al. (2020) found that gross primary production (GPP) in a semi-arid grassland was reduced more by drought than by a heatwave. However, Gharun et al. (2020) showed very different responses to the 2018 summer drought (as compared to the previous two years) among temperate grasslands at different elevations, with annual GPP decreasing at lower elevations but increasing at the alpine elevation due to abundant soil water after snowmelt. Thus, our understanding of the response of grasslands to a compound extreme drought and heatwave is controversial, particularly for alpine grasslands.

Dew was widely observed across arid (Uclés et al., 2013), temperate (Jacobs et al., 2006) and tropical (Clus et al., 2008) ecosystems, and can contribute up to 0.7–0.8 mm of water per day (Beysens, 2018). Dew amounts were quantified by lysimeters (Riedl et al., 2022), eddy-covariance (Jacobs et al., 2006), and isotopic (Kim and Lee, 2011) approaches. Nocturnal dew formation and its evaporation in the early morning hours is expected to alleviate drought and heat stress imposed by a compound extreme event due to the following reasons: (1) Dew formation is driven by radiative cooling of plant canopies with stronger long-wave outgoing radiation than that of atmospheric air on clear and calm nights (Oke, 1970), hence relieving canopy heat stress. (2) High humidity conditions under dew formation and dew water films covering foliage reduce plant transpiration (Gerlein-Safdi et al., 2018). (3) Foliar uptake of dew droplets or atmospheric water vapor alleviates plant water stress (Boucher et al., 1995; Dawson and Goldsmith, 2018). (4) Leaf gas exchange during the morning hours — when dew evaporates — might be highly relevant to alleviate plant stress, since transpiration and photosynthesis during most of the day are strongly impaired during a compound heat-drought event (Gharun et al., 2020). Oliveira et al. (2021) pointed out that evaporation of dew induced CO_2_ loss of a maritime pine forest during the rain-free period after wildfire. On the contrary, Simonin et al. (2009) reported that carbon gain was improved by fog which alleviated leaf water deficit, and addressed that the effect of dew/fog on net ecosystem exchange can vary by the duration of canopy wetting and the wettability of the leaf surface. However, it remains to be shown whether dew alleviates negative effects of a combined heat-drought on plant water status and ecosystem carbon exchange of grasslands, and which of the above mechanisms might dominate such a response.

Leaf water isotope signatures (δ^18^O and δ^2^H) are useful to assess this question because they are natural tracers that can be used to assess plant physiological responses to environmental conditions (Bachmann et al., 2015; Prechsl et al., 2015). Evapotranspiration causes ^18^O enrichment in leaf water compared to the source water (Dongmann et al., 1974; Farquhar and Lloyd, 1993). The magnitude of leaf water ^18^O enrichment is strongly affected by the isotope signal of water vapor (Cernusak et al., 2002) and dew/fog (Kim and Lee, 2011; Goldsmith et al., 2017; Gerlein-Safdi et al., 2018), but also affected by foliar transpiration rates (Gessler et al., 2013). Typically, the leaf water ^18^O signal is transferred onto leaf sugars *via* photosynthetic processes during daytime (Brandes et al., 2006; Gessler et al., 2013), and *via* the non-photosynthetic oxygen isotope exchange between leaf water and carbonyl groups of sugars (Wang et al., 2021). Leaf sugars are typically more enriched in ^18^O compared to the leaf water due to the isotopic fractionation occurring during carbonyl hydration (Yakir and Deniro, 1990). Chamber experiments also suggested the transfer of the isotope signal of dew/fog on leaf water isotope signal during light and dark conditions (Kim and Lee, 2011; Gerlein-Safdi et al., 2018; Lehmann et al., 2020), and on leaf sugars during daytime conditions (Lehmann et al., 2020). However, it is not clear how large the photosynthetic and non-photosynthetic isotope imprints of leaf water on sugars are during nighttime as well as under low light and temperature conditions in the field.

Therefore, the main goals of this study focus on these three aims:

1) Quantify the combined effects of heat-drought stress and dew on net ecosystem production (NEP) by comparing the NEP before and during the heatwave, and analyzing leaf water-sugar isotope exchange in a chamber tracer experiment.
2) Quantify the combined effect of heat-drought stress and dew on plant water status by physiological and water isotope measurements.
3) Identify controls of atmospheric and soil conditions on plant water *via* analyzing the correlations of environmental variables with plant physiological and isotopic indicators.

We addressed these three aims using field data collected at an alpine grassland before and during the June 2019 heatwave, when a combined daytime heat-drought stress for the vegetation occurred during the day and dew formed during the night. H_2_O vapor and net ecosystem CO_2_ exchange were measured with the eddy-covariance (EC) technique to assess the effects of these environmental conditions on the vegetation at the ecosystem scale. Physiological and water isotope measurements were employed to analyze the response of vegetation to these environmental conditions at plant scales.

## 2 Materials and methods

### 2.1 Study site

**Figure 1:**
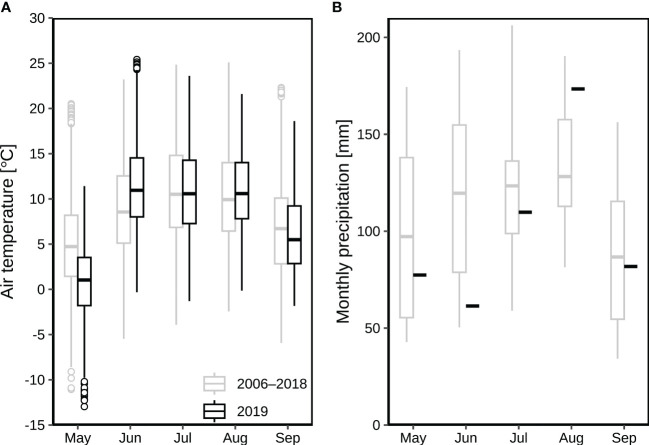
Air temperature and precipitation at the alpine grassland site CH-AWS in 2019 as compared to the period 2006–2018 (all data collected at the site): **(A)** average monthly air temperatures from May to September; **(B)** monthly precipitation from May to September. The boxplots show the medians, 25, and 75 quantile values (the inter-quartile range, IQR), with whiskers showing the data range up to 1.5 times the IQR. Values outside that range are shown with symbols.

The Alp Weissenstein research site (CH-AWS, at 2000 m.a.s.l.) is part of a managed (grazed) alpine grassland ranging from 1900 to 2500 m.a.s.l. The vegetation composition was classified as *Deschampsio cespitosae*–*Poetum alpinae* community with red fescue (*Festuca rubra*), Alpine cat’s tail (*Phleum rhaeticum*), white clover (*Trifolium repens*) and dandelion (*Taraxacum officinale*) as dominant species (Keller, 2006), complemented by alpine meadow-grass (*Poa alpina*) and lady’s mantle (*Alchemilla vulgaris*). The soil types are slightly humous to humous sandy loam (Hiller et al., 2008), hence the permanent wilting point is estimated at around 0.1 m^3^ m^-3^. The mean annual air temperature and precipitation were 1.9 °C (2015–2020; measured all year round at the site between 2015 and 2020; before 2015, data were only collected between May and October at the site) and 1213 mm (2013–2020; measured all year round between 2013 and 2020; before 2012, only the liquid precipitation was measured at the site), respectively. During the main growing season (May to September), monthly mean air temperatures were between 5.0 °C and 10.8 °C (2006–2018) with July as the hottest month (**Figure 1A**), while average monthly precipitation ranged from 87 to 128 mm (**Figure 1B**). During the growing season in 2019, the monthly temperature ranged from 0.8 °C to 11.7 °C with June as the hottest month (**Figure 1A**), whilst the monthly precipitation ranged from 61 mm to 173 mm (**Figure 1B**).

### 2.2 Eddy covariance and meteorological measurements

EC measurements for H_2_O vapor and net ecosystem CO_2_ exchange have been carried out during the growing season since 2006 (tower coordinates: 46°34’59.5” N, 9°47’25.5” E at 1978 m.a.s.l.). In mid-November 2014, the site was equipped with mains power for year-round operation. The EC instruments at CH-AWS in 2019 consisted of a three-dimensional sonic anemometer (model HS-50, Gill Instruments, Solent, UK) and an enclosed-path infrared gas analyzer (IRGA; Li-7200, Li-Cor, Lincoln, NB, USA), installed at 1.4 m agl (above ground level). EC measurements were recorded at 20 Hz and processed to 30 min averages using the EddyPro software Version 7.0.6 (LI-COR, 2019) following established community guidelines (Aubinet et al., 2012) for H_2_O (*F*_H_2_O_ in mmol m^-2^ s^-1^) and CO_2_ fluxes (*F*_CO_2__ in μmol m^-2^ s^-1^; net ecosystem exchange NEE). The micro-meteorological sign convention was used, with negative values denoting a downward flux, while positive values stand for upward fluxes. See details of footprint analysis of eddy-covariance measurements in Zeeman et al. (2010). Vapor pressure deficit (VPD) was quantified for 30-min intervals from ancillary air temperature and relative humidity measurements at 1.4 m agl (HygroClip HC2, Rotronic, Bassersdorf, Switzerland). Photosynthetic photon flux density (PPFD in μmol m^-2^ s^-1^) was measured at 1.3 m agl every 10 s (PARlite, Kipp & Zonen B.V., Delft, The Netherlands) and then averaged to 30-min intervals. Volumetric soil water content (SWC) was measured by two sensors (EC-5, Decagon Devices, Inc., Pullman, WA, USA) at 5 cm depth. NEP was calculated with the opposite sign of NEE (NEP = –NEE).

Diurnal NEP (g C m^-2^) was calculated from the CO_2_ flux ( $F_{CO_{2}}$ ):


(1) $$\text{NEP}=\sum \left(a\cdot t\cdot F_{{\text{CO}}_{2}}\cdot M_{\text{C}}\right)$$

where *M*_c_ is the molar mas of carbon (12 g C mol^-1^), *t* is measurement intervals (1800 s) of $F_{CO_{2}}$ , and a is a unit conversion factor (10^-6^ mol μmol^-1^).

For a long-term data series of air temperature at standard 2 m agl, an additional meteorological measurement setup was operating since 2006, installed at about 1180 m distance to the east and at approximately 40 m higher elevation compared to the flux tower (Michna et al., 2013). This additional setup provided air temperature (*T*_a_) at 2 m agl (shaded, sheltered HydroClip S3, Rotronic AG, Basserdorf, Switzerland), and precipitation from an unheated pluviometer (LC, Texas Electronics, Dallas, USA). In November 2012, a precipitation gauge (1518H3, LAMBRECHT meteo GmbH, Göttingen, Germany) with a heatable orifice was installed between these two measurement stations (Michna et al., 2013) and provided annual total precipitation, including snowfall. Leaf wetness data were averaged from the measurements of two leaf wetness sensors (BNS, G. Lufft Mess-und Regeltechnik GmbH, Fellbach, Germany) installed since 2005 at 0.1 m agl close to the grassland canopy, using blotting paper inside a clip holder. The leaf wetness data was recorded by the voltage signal resulting from a fixed current applied from the center to the rim of the blotting paper. When the paper got wet by dew, fog, or rain, the blotting paper became conductive, and an increase in voltage signal was observed. We note that the leaf wetness sensors overestimated the leaf wetting duration (**Figure S2**), because the blotting paper dries out slower than the vegetation. By comparing the BNS sensor with a more accurate leaf wetness sensor (PHYTOS 31, Meter Group AG, Munich, Germany) at a later time of our observation campaigns (5–6 July 2020), the termination of leaf wetting was defined as the point when leaf wetness by BNS steeply and linearly decreased (**Figure S2**).

All variables were aggregated to 30 min averages or sums. The time series was recorded in CET (UTC+1 hour).

The evapotranspiration rate (ET in mm h^-1^) was calculated from the H_2_O flux ( $F_{H_{2}O}$ ) as (Stull, 1988):


(2) $$\text{ET}\;=b\;\cdot \;F_{{\text{H}}_{2}\text{O}}\;\cdot \;M_{{\text{H}}_{2}\text{O}}$$

where $M_{H_{2}O}$ is the molar mass of H_2_O (18 g mol^-1^), and *b* is a unit conversion factor [= (10^-3^ mol mmol^-1^) · (10^-6^ m^3^ g^-1^) · (3600 s h^-1^) · (10^3^ mm m^-1^) = 0.0036 mol m^2^ s mm mmol^-1^ g^-1^ h^-1^].

### 2.3 June 2019 heatwave and drought

According to our measurements at the CH-AWS site during 2006 to 2018, the hottest three months were typically June, July, and August, with average air temperatures (at standard 2 m agl) of 8.9, 10.8 and 10.3 °C (**Figure 1A**), respectively. As compared to the long-term averages, the respective three months in 2019 were hotter with 11.7, 10.9, 10.8 °C (**Figure 1A**). The precipitation in June 2019 was 61 mm, which was only 51% of the long-term average of 120 mm in June during 2006–2018 (**Figure 1B**).

The 2019 heatwave occurred in Switzerland from 25 June to 1 July in 2019 (MeteoSwiss, 2019). No rain was recorded at the site from 23 June to 30 June 2019, but 0.2 mm rain was collected at 16:00 CET on 1 July 2019. Therefore, in this study, we only considered the 8-day rain-free period between 23 and 30 June 2019, with 23–24 June before the heatwave, and 25–30 June during the heatwave. Sunrise was around 04:30, and sunset was around 20:20 during the heatwave.

### 2.4 Experimental setup during measurement campaigns

To assess the combined effect of a well-developed natural drought during the heatwave in June 2019, we conducted two intensive measurement campaigns as intensive observation periods (IOP) at the end of the heatwave. These campaigns were carried out during two consecutive dew nights on 28–29 (IOP1 from 12:00 to 12:00 the next day) and on 29–30 (IOP2) June 2019.

#### 2.4.1 Destructive sampling for isotope composition of water samples

To measure the isotope composition (δ^18^O and δ^2^H) of leaf water, xylem water of root crowns, soil water, and dew droplets on leaf surfaces, destructive sampling was carried out during the IOP within 1 h before sunset (19:30 of IOP1 and IOP2), during the night (00:00 and 03:00 of IOP1 and IOP2), and after sunrise (06:00 of IOP1). Bulk leaf samples were taken in triplicates from randomly selected plants of four genera within an area of 70×20 m^2^, i.e., *Alchemilla* with palmately-lobed and hairy leaves, as well as toothed leaf edges; *Poa* with long and narrow grass leaves; *Taraxacum* with a rosette of long and wide jagged leaves; and *Trifolium* with obovate leaves (**Figure S1**). The average vegetation height was around 20 cm during our field campaigns. Root crown xylem samples were taken in triplicates after removing the attached soil and debris from randomly selected plants for each genus. Dew droplets were absorbed with cotton balls in six replicates from randomly selected plants. Soil cores were taken with a soil auger in triplicates and were then cut into slabs to separate four soil depths of 0–5 cm, 5–10 cm, 10–15 cm, and 15–20 cm. Leaf samples were taken at 19:30, 00:00, 03:00 and 06:00 during IOP1, as well as at 19:30, 00:00 and 03:00 during IOP2. Root crown samples were taken at 19:30, 00:00 and 03:00 during IOP1 and IOP2. Dew droplets were taken at 03:00 during IOP1 (no dew droplets were observed at 00:00), as well as at 00:00 and 03:00 during IOP2. The 0–5 cm soil samples were taken at 19:30, 00:00 and 03:00 during IOP1 and IOP2, while soil samples of 5–10 cm, 10–15 cm and 15–20 cm depth were taken at 19:30 and 03:00 during IOP1 and IOP2.

All samples were immediately transferred into glass tubes (Labco Exetainer^®^ 12 ml Vial, Labco Ltd., Lampeter, UK), sealed with caps and parafilm, and stored in a portable freezing box filled with dry ice blocks. Samples were then taken back to the laboratory and stored at –19 °C. Dew water from cotton balls, and water from all plant and soil samples were extracted using a cryogenic vacuum extraction system (Prechsl et al., 2015). Using the high-temperature carbon reduction method (Werner and Brand, 2001; Gehre et al., 2004), the isotope composition of the respective water samples was determined by an isotope ratio mass spectrometer (IRMS, DeltaplusXP, Finnigan MAT, Bremen, Germany) coupling with a high-temperature conversion elemental analyzer (TC/EA, Finnigan MAT, Bremen, Germany) *via* a ConFlo III reference unit (Finnigan MAT, Bremen, Germany). The precision of δ^18^O and δ^2^H measurements for all the samples was ±0.3‰ and ±0.7‰, respectively. All isotope values of this study are expressed in the delta notation δ=(*R*_sample_/*R*_standard_–1) in per mil (‰), where *R*_standard_ and *R*_sample_ are the molar ratios of either ^2^H/^1^H or ^18^O/^16^O of the standard (Vienna Standard Mean Ocean Water, V-SMOW) and the sample (IAEA, 2009; Coplen, 2011).

#### 2.4.2 Isotope composition of atmospheric water vapor

The atmospheric water vapor at around 1 m agl was collected during IOP2 from 20:30 to 23:30, and from 00:00 to 03:00. Atmospheric air was pulled through a U-shaped glass tube that was placed in a Dewar filled with a cold slurry of ethanol and dry ice. After 3 h, the trapped water vapor frozen to the inner walls of the U-shaped glass tube was thawed, and the liquid water was filtered (Syringe filter, PTFE-Hydrophobic, 0.45μm) and transferred into glass vials. The samples were measured with the TC/EA-IRMS for their isotope composition (δ^18^O_vapor_ and δ^2^H_vapor_) as described above. To compare the isotope composition of atmospheric water vapor with the liquid water pools (i.e., dew droplets, leaf water, xylem water of root crowns, and soil water), the isotope composition of the liquid (δ^18^O_eq_ and δ^2^H_eq_) in equilibrium with this vapor was calculated under the corresponding air temperature measured at 1.4 m agl following Horita and Wesolowski (1994).

#### 2.4.3 Leaf water potential

To investigate the mechanism of dew influence on ecosystem water and carbon exchanges, leaf water status was measured at the end of the heatwave during our intensive observation campaigns (IOP1 and IOP2). The comparison of LWP before and after 29 June heatwave as well as two dew nights (the 28–29 and 29–30 nights) allows to compare the influence of heatwave and dew on leaf water status.

Leaf water potential (LWP) of the four genera *Alchemilla*, *Poa*, *Taraxacum*, and *Trifolium* was measured in triplicates with a Scholander pressure chamber (Model 1505D, PMS Instruments Co., Albany, OR, USA) using a grass compression gland for *Poa* and *Taraxacum*, and a round compression gland for *Alchemilla* and *Trifolium*. The LWP was measured within 1 h before sunset (19:30; before-sunset) and 2 h before sunrise (03:00; predawn) during IOP1 and IOP2.

#### 2.4.4 Complementary *in-situ* chamber tracer experiment

Complementary to the sampling campaigns under natural conditions, an *in-situ* chamber tracer experiment was carried out during IOP1 (28–29 June) to investigate whether dew signal was used for carbon assimilation by determining the δ^18^O and δ^2^H values of leaf water and sugars. Within around 1 h before sunset, a 50 × 50 cm^2^ grassland plot was marked for the chamber tracer experiment. We used liquid water depleted in ^18^O and ^2^H as tracer (δ^18^O = –364.7 ± 1.9‰ and δ^2^H = –775.0 ± 0.3‰) and homogenously sprayed it onto the plot at around 19:30. After spraying, the vegetation was immediately covered with a custom-made canopy chamber (50 × 50 × 50 cm^3^) wrapped with 0.1 mm thick polyethylene film, with a 76% transmissivity for thermal (longwave) infrared radiation (Horiguchi et al., 1982). With this tracer addition, we simulated dew, which was much more depleted in ^18^O and ^2^H than natural dew droplets. We note that we did not isolate the soil during the tracer amending on the grassland plot, hence the tracer could drip into the soil, and the amended tracer on vegetation can also drip to the soil, both of which can occur during natural dew formation processes. The canopy chamber did not fully isolate the grassland from the surrounding; thus, gas emission could still occur from the bottom rim of the chamber. But the chamber sufficiently suppressed the water vapor exchange between the within-chamber air and the open atmosphere. About 2 h before (03:00; predawn) and after sunrise (06:00), bulk leaf samples were taken in triplicates from randomly selected plants per genus in the plot. In addition, bulk leaf samples taken before sunset (19:30) acted as control for this experiment. Leaf water was extracted for isotope analyses (δ^18^O_lw_ and δ^2^H_lw_) as described in Section 2.3.1.

Leaf dry matter after cryogenic water extraction was milled to fine powder for δ^18^O_ls_ and δ^13^C_ls_ analysis of leaf sugars. Bulk sugars were extracted from 60 mg of this leaf powder with 1.5 mL deionized water at 85 °C for 30 min (Lehmann et al., 2020). The neutral sugar fraction (defined here as “sugars”) was then further purified from ionic and phenolic substances by ion-exchange cartridges (OnGuard II A, H and P, Dionex; Thermo-Fisher Scientific, Bremen, Germany) following the protocol by Rinne et al. (2012). For the analysis of δ^18^O_ls_, the purified bulk leaf sugars were filled into silver capsules, frozen and freeze-dried. The measurement precision (standard deviation) of the quality control standard (cellulose with 27.6‰ for δ^18^O) was ≤ 0.3‰ for δ^18^O_ls_ (Lehmann et al., 2018), and 0.1‰ for δ^13^C_ls_ (Bögelein et al., 2019). δ^13^C is the carbon isotope ratio in δ-notation in per mill (‰), relative to the international Vienna-Pee Dee Belemnite (V-PDB) standard, and were normalized by IAEA-CH7 (polyethylene, −32.2‰) and IAEA-CH3 (cellulose, −24.7‰) (Bögelein et al., 2019). Higher δ^13^C_ls_ indicated lower water use efficiency (WUE) of plants.

In the chamber tracer experiment, the contribution of the tracer (*f*_tracer_) to leaf water at 03:00 (δ^18^O_lw_03:00_) was simulated using a linear two-pool mixing model. One source of the leaf water was assumed to be the δ^18^O_root_ at 03:00 of IOP1 (δ^18^O_root, 03:00_) under natural conditions and the second source was the mean tracer δ^18^O (δ^18^O_tracer, mean_) taken up by the leaves during the night. We calculated δ^18^O_tracer, mean_ as the mean of the original tracer δ^18^O (δ^18^O_tracer, 19:30_ = –364.7 ± 1.9‰) and the δ^18^O of the tracer which remained on the leaf surfaces by 03:00 (δ^18^O_tracer, 03:00_ measured by absorbing the remaining tracer in form of simulated dew from the leaf surfaces at 03:00). *f*_tracer_ was calculated as:


(3) $$f_{\text{tracer}}\;=\;\frac{\delta^{18}{\text{O}}_{\text{lw},\;03:00}\;-\;\delta^{18}O_{\text{root},\;03:00}}{\delta^{18}{\text{O}}_{\mathrm{tracer},\;\text{mean}}\;-\;\delta^{18}{\text{O}}_{\text{root},\;03:00}}$$

Due to the chamber acting as a heat-trap, the within-chamber temperature should be slightly higher than the open-air temperature. This temperature difference might affect the leaf-air water vapor exchange, but was assumed to have minor effect on foliar water uptake of liquid-phase dew and water-sugar isotope exchange.

### 2.5 Statistics

Tukey’s honest significance test was used to assess differences among averages over sampling times and genera by the R-function agricolae::HSD.test (Steel, 1997) and one-way ANOVA. Reported statistical significance represents *p* < 0.05 with capital letters indicating temporal differences, and lower-case letters denoting genera or soil-depth differences. The isotopic and LWP results were reported in mean and standard errors of mean (SEM). We note that differences of isotope composition are always reported in absolute terms in per mil (‰). Correlation coefficients of regressions of NEP with *T*_a_, PPFD, and RH were analyzed before and during the heatwave, with “***”, “**”, “*” and “ns” indicating *p* < 0.001, *p* < 0.01, *p* < 0.05, and *p* ≥ 0.05, respectively. During IOP1 and IOP2 at the end of the heatwave, considering the individual variability of plants, median values of leaf water isotope (δ^18^O_lw_) and LWP by species at each sampling time were used for analyzing their correlations with environmental conditions (RH, SWC, and δ^18^O_soil_), with “***”, “**”, “*” and “ns” indicating *p* < 0.001, *p* < 0.01, *p* < 0.05, and *p* ≥ 0.05, respectively. All analyses were carried out with R version 4.1.2 (R Core Team, 2021).

## 3 Results

### 3.1 Diel environmental variability before and during the heatwave

**Figure 2:**
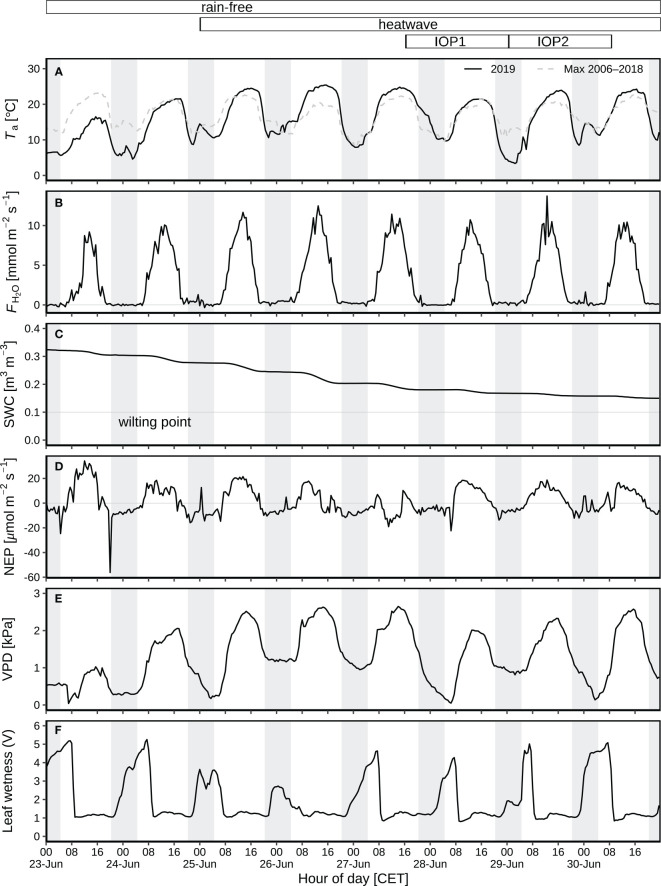
Environmental variables during the rain-free period (23–30 June) at CH-AWS site before the heatwave on 23–24 June, and during the June 2019 heatwave on 25–30 June: **(A)** air temperature (*T*_a_) at 2 m agl as compared to the long-term maximum 2006–2018. **(B)** Eddy-covariance H_2_O flux. **(C)** Volumetric soil water content (SWC) at 5 cm depth. **(D)** Net ecosystem production (NEP); positive numbers indicate CO_2_ emission, and negative numbers represent CO_2_ uptake. **(E)** Vapor pressure deficit (VPD). **(F)** Leaf wetness recorded by the voltage signal resulting from a fixed current applied from the center to the rim of the blotting paper; substantial increase in leaf wetness during nighttime and early morning indicate leaf wetting by dew during the rain-free period; dew occurred on each night of the period. Field campaigns were carried out during two intensive observation periods (IOP) on 28–29 (IOP1) and 29–30 (IOP2) June 2019. Hours of day are given in CET. The grey shaded areas represent nocturnal periods.

**Figure 3:**
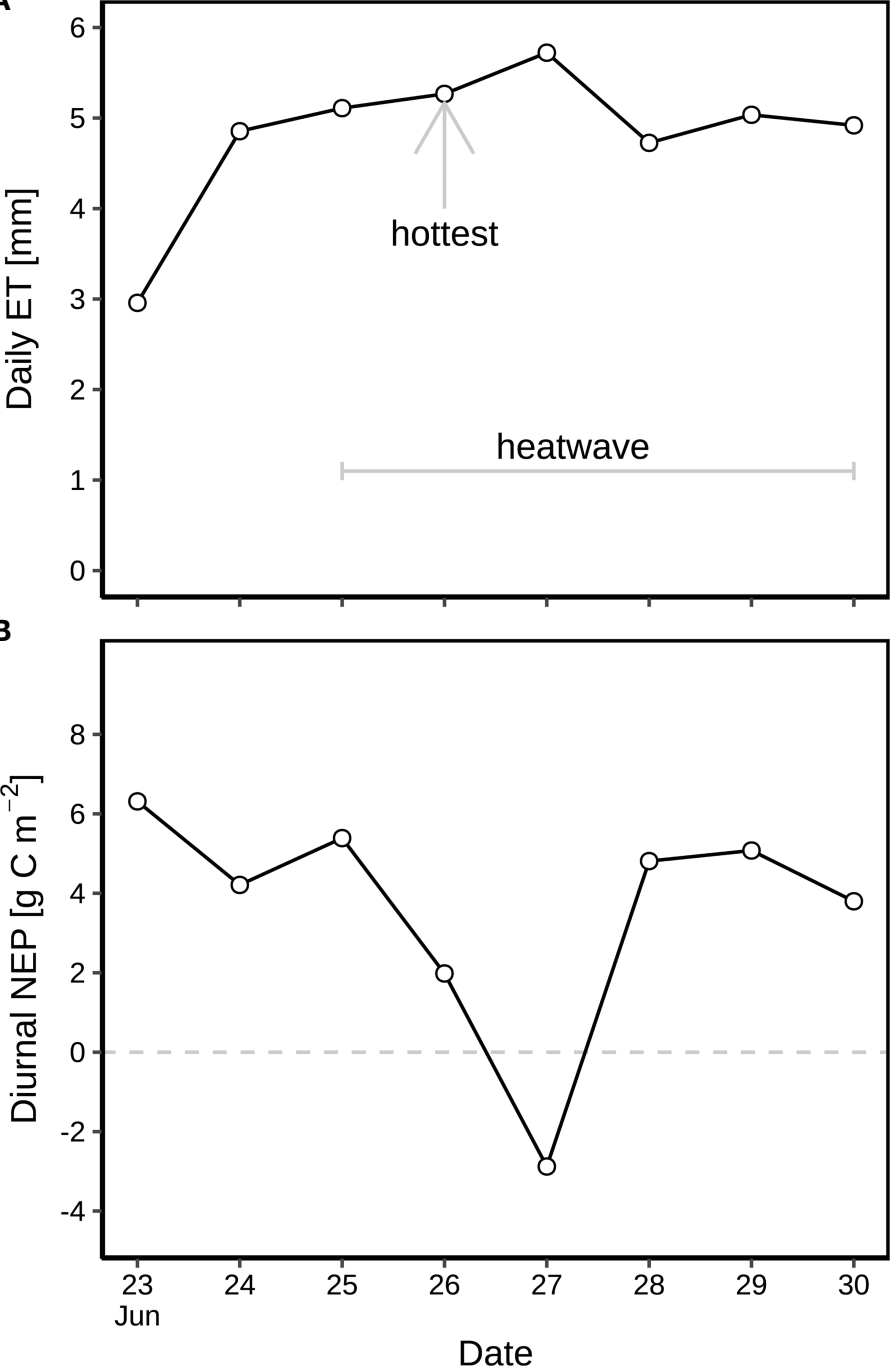
**(A)** Diurnal evapotranspiration (ET) and **(B)** net ecosystem production (NEP) during the rain-free period (23–30 June) at CH-AWS site before the heatwave on 23–24 June, and during the June 2019 heatwave on 25–30 June. Dew occurred on each night of the period. 26 June was hottest during the heatwave.

The diurnal and nocturnal air temperature averaged 14.5 °C and 7.3 °C before the heatwave during 23-24 June, but was 20.1 °C and 11.2 °C on average during the heatwave from 25 to 30 June (**Figure 2A**). The highest temperature of 25.4 °C was observed on 26 June 2019 (15:30; **Figure 2A**), indicating the peak of the heatwave, followed by 27 June 2019, the second hottest day. H_2_O fluxes varied from –0.3 to 13.7 mmol m^-2^ s^-1^ (**Figure 2B**), corresponding to 3.0–4.7 mm of diurnal ET before the heatwave, and 4.9–5.7 mm during the heatwave (**Figure 3A**). SWC decreased from 0.32 to 0.15 m^3^ m^-3^ during this rain-free period from 23 to 30 June (**Figure 2C**). NEP varied from –22 to 21 μmol m^-2^ s^-1^ (**Figure 2D**). The daytime NEP was 4.2–6.3 g C m^-2^ before the heatwave, but ranged from 5.4 to –2.9 g C m^-2^ during the heatwave (**Figure 3B**). Negative NEP (–2.9 g C m^-2^) occurred on 27 June, the day after the hottest day (26 June). Highest VPD was 2.06 kPa and 2.65 kPa before and during the heatwave (**Figure 2E**), respectively. The leaf wetness levels indicated that dew occurred during each night of the rain-free period, being fully evaporated from vegetation surfaces after 07:30 of day (**Figure 2F**). With dew occurrence during each night of the rain-free period (23–30 June), the corresponding nocturnal VPD was as low as 0.14–1.41 kPa (**Figure 2E**).

### 3.2 Effects of heat-drought and dew on net ecosystem production

**Figure 4:**
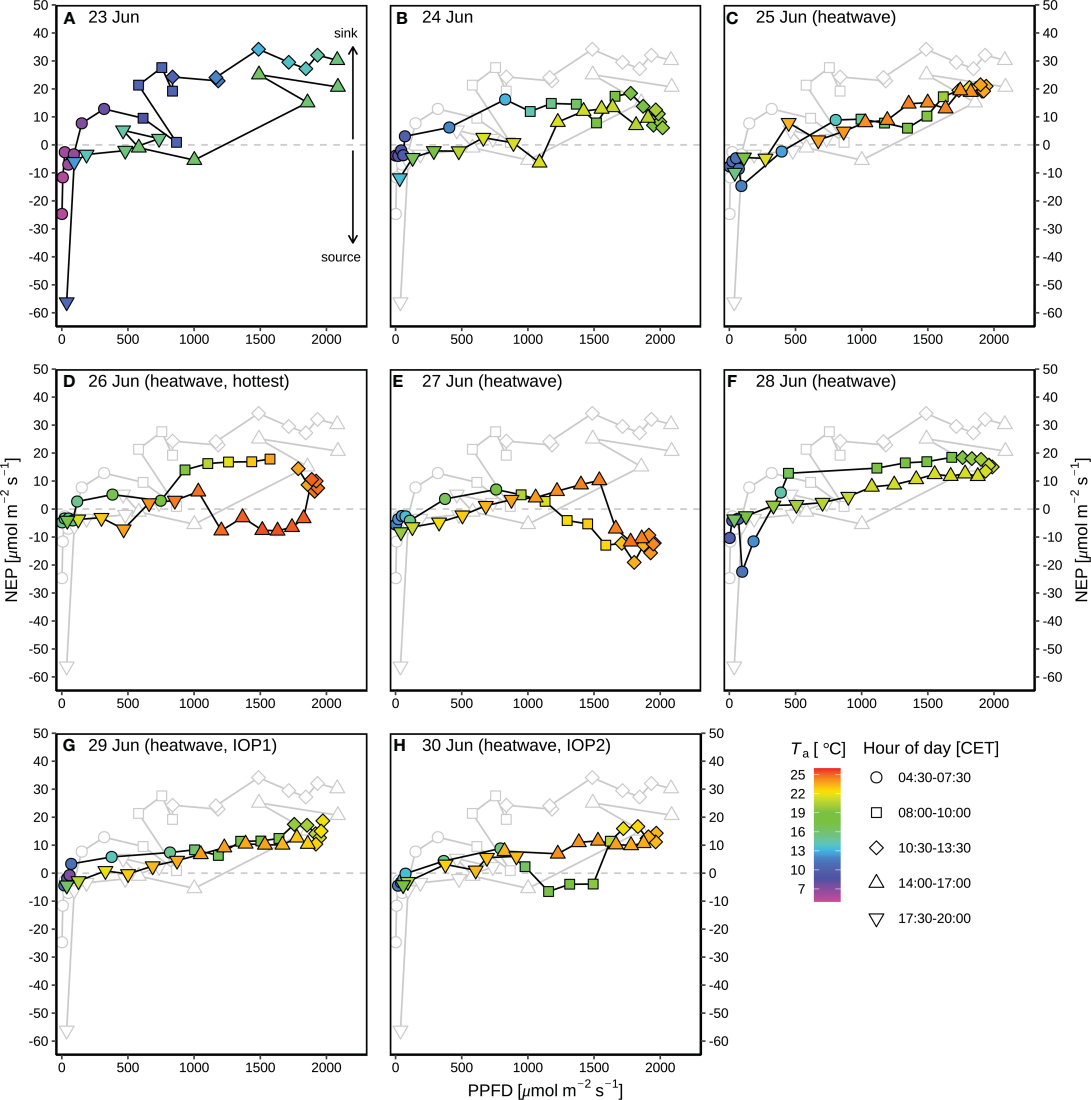
Response curves of diurnal net ecosystem production (NEP) to photosynthetic photon flux density (PPFD) during the rain-free period (23–30 June) at CH-AWS site before the heatwave on 23–24 June **(A, B)**, and during the June 2019 heatwave on 25–30 June **(C–H)**. Different hours of day (CET) are shown in different shapes; the colors of the symbols indicate the corresponding air temperature (*T*_a_). Dew occurred during each night of the period. For comparison, the grey plots in panels **(B–H)** show the NEP-PPFD response curve on 23 June 2019 before the heatwave.

Before the heatwave, NEP almost linearly increased with PPFD (**Figure 4**), with higher diurnal NEP (6.3 g C m^-2^) on 23 June than that on 24 June (4.2 g C m^-2^; **Figure 3B**). The suppression of heat-drought stress on NEP was substantial on the hottest day (26 June with 2.0 g C m^-2^) during the heatwave (**Figure 3B**), with 63% of reduction in NEP compared to the previous day (25 June with 5.4 g C m^-2^ of NEP). Negative diurnal NEP (–2.9 g C m^-2^) occurred on the second hottest day (27 June; **Figure 3B**), with longer period (09:00–15:00) of net carbon emission (negative NEP) under PPFD > 1300 μmol m^-2^ s^-1^ and *T*_a_ > 22.5 °C (**Figure 4E**), as compared to the hottest day during 14:00–16:30 under PPFD > 1800 μmol m^-2^ s^-1^ and *T*_a_ > 25.3 °C (**Figure 4D**). Daily NEP recovered to the levels of 4.7–5.0 g C m^-2^ on 28–30 June after the hottest two days on 26–27 June (**Figure 3B**).

**Figure 5:**
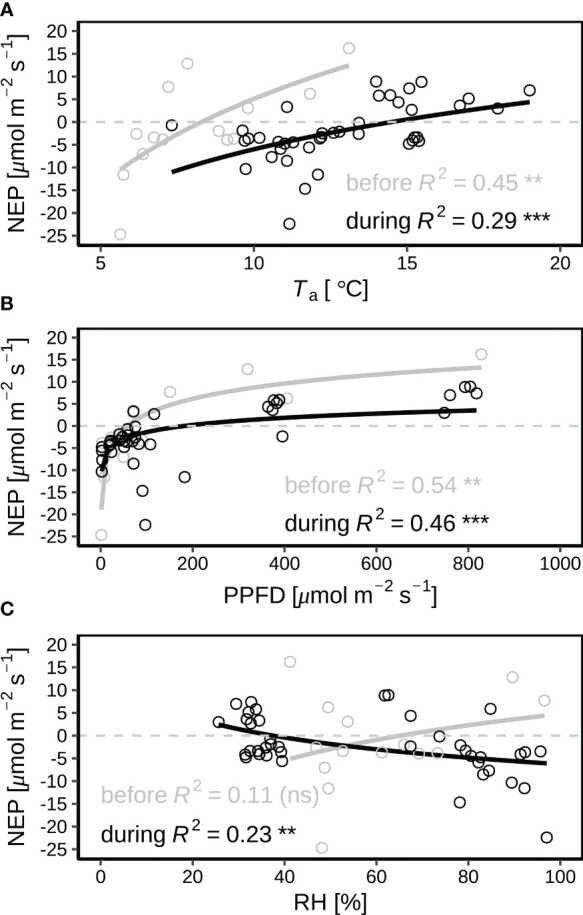
Correlations of net ecosystem production (NEP) with **(A)** air temperature (*T*_a_ at 2 m agl), **(B)** photosynthetic photon flux density (PPFD), and **(C)** relative humidity (RH) in the early morning hours (from sunrise to 07:30) with wet leaves by dew before (23–24 June in grey) and during the June 2019 heatwave (25–30 June in black). Dew occurred during each night of the period. 26 June was hottest during the heatwave. The values of *p* for the correlation coefficients are indicated by “***”, “**”, “*” and “ns” for *p* < 0.001, *p* < 0.05, *p* < 0.01, and *p* ≥ 0.05.

During the early morning hours before 07:30 with leaf wetting by dew, NEP increased by *T*_a_ before and during the heatwave (**Figure 5A**; *p* < 0.01 and *p* < 0.001 before and during the heatwave, respectively), but the turning *T*_a_ from negative (net carbon emission) to positive (net carbon sequestration) NEP was higher during the heatwave period (13.7 °C for 25-30 June) than that before the heatwave (8.2 °C for 23-24 June). With vegetation wetting by dew in the early morning hours, NEP exponentially increased with PPFD (*p* < 0.01 and *p* < 0.001 before and during the heatwave, respectively), but NEP was lower during the heatwave than that before the heatwave under same levels of PPFD (**Figure 5B**). In the early morning hours with vegetation wetting, NEP slightly increased by leaf wetness levels before the heatwave (**Figure 5C**; *p* ≥ 0.05), but significantly decreased by leaf wetness levels at the beginning of the heatwave on 25–26 June (**Figure 5D**; *p* < 0.01).

### 3.3 Effects of heat-drought and dew on leaf water status and leaf isotopes

#### 3.3.1 Leaf water status

**Figure 6:**
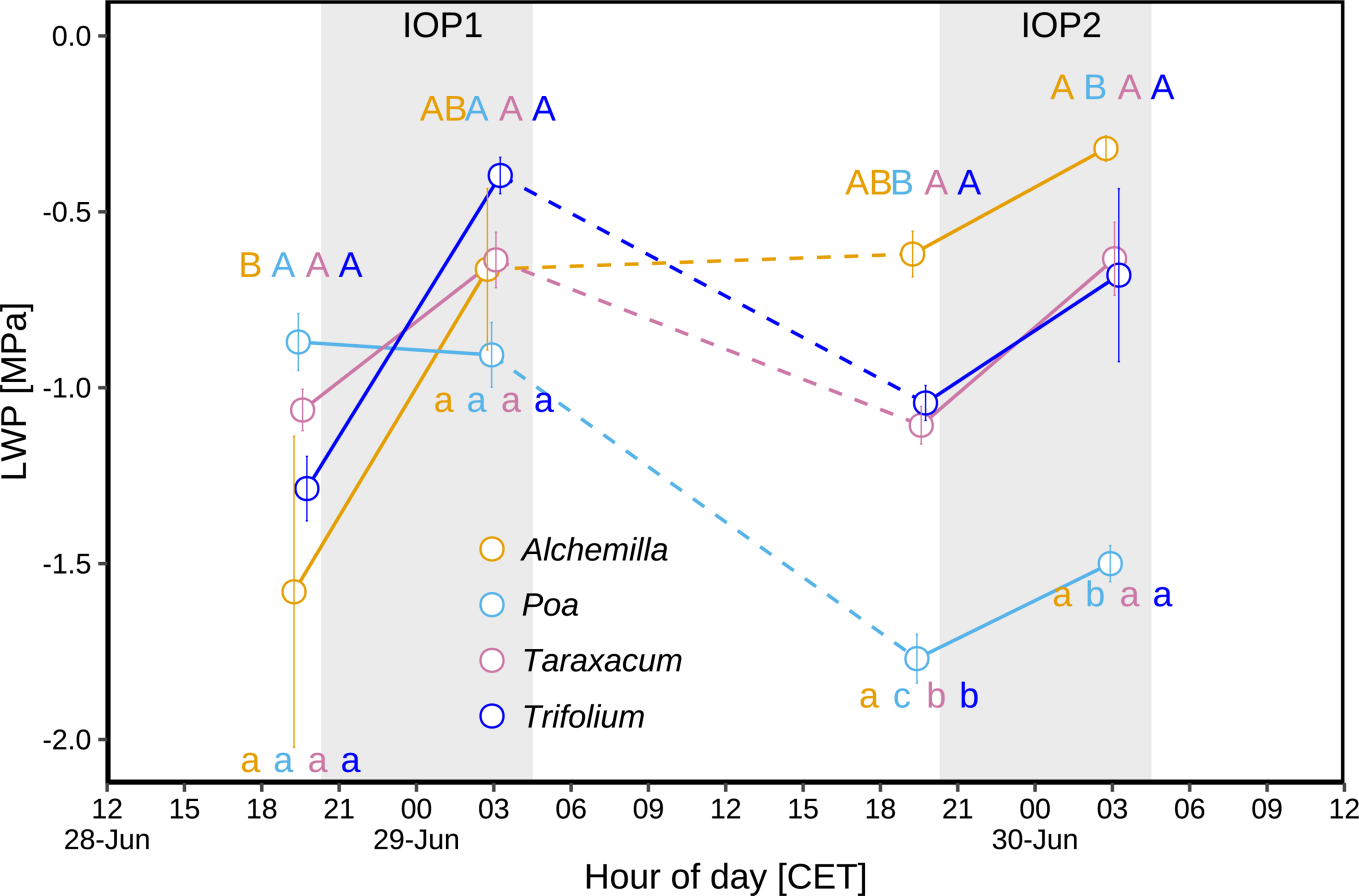
Leaf water potential (LWP) during two intensive observation periods (IOP1 and IOP2) with nocturnal dew occurrence at the end of the June 2019 heatwave: LWP of different plant genera (*Alchemilla*, *Poa*, *Taraxacum*, and *Trifolium*) were measured before sunset (at 19:30 within around 1 h before sunset) and before sunrise (03:00 within around 2 h before sunrise) of two dew events. Different letters indicate statistically significant differences (*p* < 0.05), with capital letters indicating temporal comparison, and lower cases indicating among-genera comparison. The grey shaded areas represent nocturnal periods.

Comparing predawn (03:00) periods of IOP1 and IOP2 (**Figure 6**), *Poa* LWP significantly decreased from –0.9 to –1.5 MPa (*p* < 0.05), *Trifolium* LWP slightly decreased from –0.4 to –0.7 MPa (*p* ≥ 0.05), *Taraxacum* LWP was at similar levels (–0.6 to –0.6 MPa; *p* ≥ 0.05), whilst *Alchemilla* LWP slightly increased from –0.7 to –0.3 MPa (*p* ≥ 0.05). Comparing before-sunset (19:30) periods of IOP1 and IOP2 (**Figure 6**), *Poa* LWP significantly decreased from –0.9 to –1.8 MPa (*p* < 0.05), *Taraxacum* LWP (–1.1 to –1.1 MPa) was at similar levels, whilst *Trifolium* LWP slightly increased from –1.3 to –1.0 MPa (*p* ≥ 0.05) and *Alchemilla* LWP increased from –1.6 to –0.6 MPa (*p* ≥ 0.05). The LWP of all four genera slightly increased during both IOP nights (*p* ≥ 0.05; **Figure 6**), i.e., from before-sunset (19:30) to predawn periods (03:00). Among the four genera, *Alchemilla* had the lowest LWP before sunset of IOP1 (*p* ≥ 0.05), but had slightly higher LWP than the other three genera before sunset of IOP2 (*p* ≥ 0.05). On the contrary, *Poa* had slightly lower LWP than the other three genera at the predawn period of IOP1 (*p* ≥ 0.05), but had much lower LWP than the other three genera at predawn of IOP2 (*p* < 0.05). Therefore, *Poa* LWP significantly decreased (*p* < 0.05) at the end of the heatwave, whilst *Alchemilla* LWP significantly increased (*p* < 0.05), *Taraxacum* and *Trifolium* slightly increased (*p* ≥ 0.05) although heat-drought stress.

#### 3.3.2 Isotope composition of different water pools at natural isotope abundances

**Figure 7:**
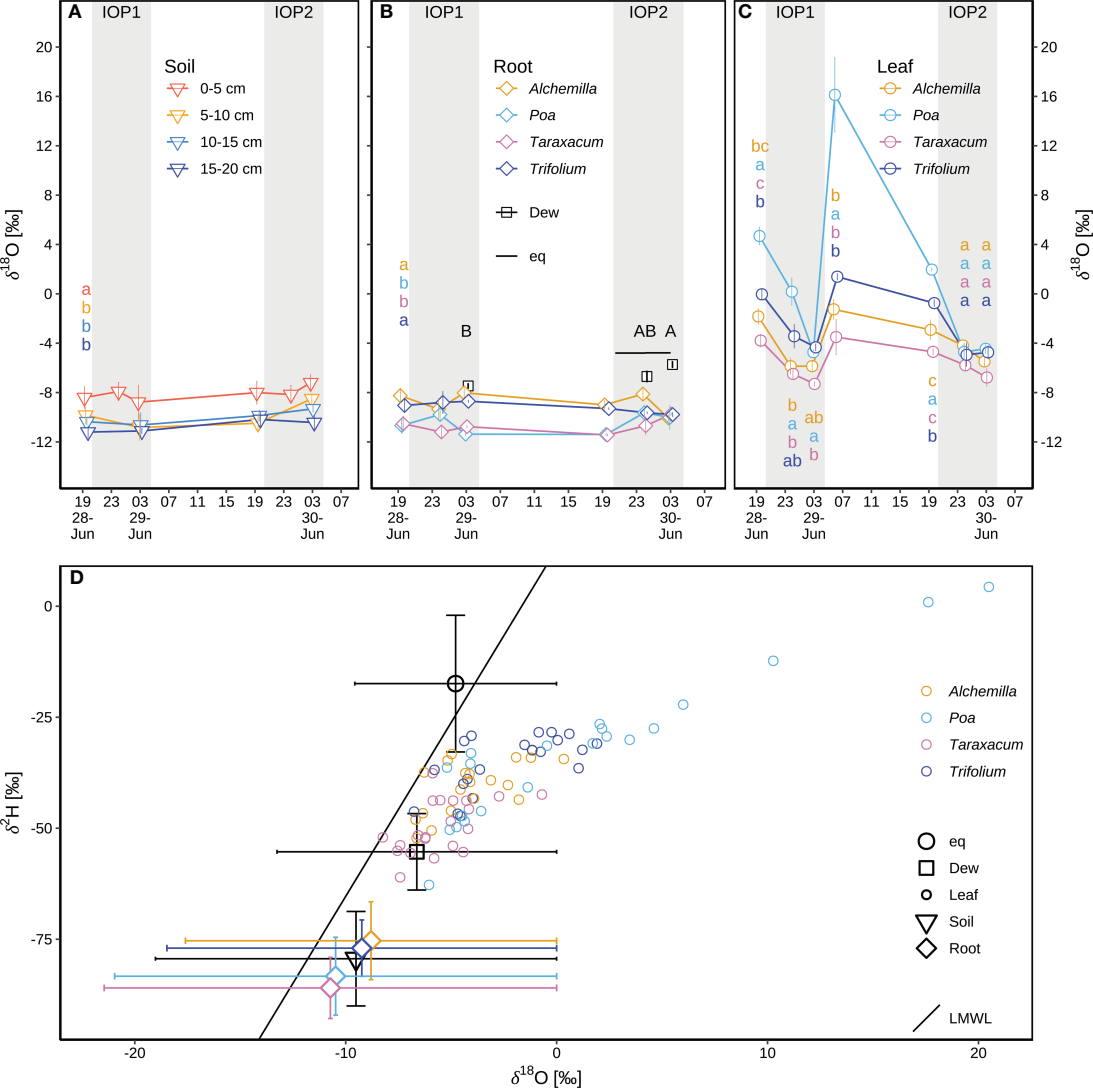
Isotope composition of different water pools during two consecutive intensive observation periods (IOP1 and IOP2) with nocturnal dew formation at the end of the June 2019 heatwave: **(A)** soil water δ^18^O_soil_ at different depths (0–5, 5–10, 10–15, and 15–20 cm); different lower-case letters indicate statistical significance of among-soil-depth difference (*p* < 0.05) of δ^18^O_soil_ over IOPs. **(B)** Dew droplets δ^18^O_dew_ on plant surfaces; the liquid δ^18^O_eq_ in equilibrium with atmospheric water vapor; xylem water of root crown δ^18^O_root_ for four genera (*Alchemilla*, *Poa*, *Taraxacum*, *Trifolium*); different lower-case letters indicate statistical significance of among-genera difference (*p* < 0.05) of δ^18^O_root_ over IOPs; different capital letters in black indicate statistical significance of among-sampling-time difference (*p* < 0.05) of δ^18^O_dew_. **(C)** Leaf water δ^18^O_lw_ for four genera; different lower-case letters indicate statistical significance of among-genera difference (*p* < 0.05) for δ^18^O_lw_ at each sampling time. Mean and standard errors of mean (SEM) are shown in panels **(A–C)**. **(D)** δ^2^H–δ^18^O pairs compared to the local meteoric water line (LMWL: δ^2^H = 7.83δ^18^O + 12.97) following Prechsl et al. (2014); δ^2^H–δ^18^O of equilibrium liquid, dew samples, and soil samples was mean and SEM over IOPs; δ^2^H–δ^18^O of xylem water of root crown was mean and SEM by species over IOPs; raw data of leaf water δ^2^H–δ^18^O were shown for four genera at each sampling time. The grey shaded areas in panels **(A-C)** represent nocturnal periods.

The δ^18^O_soil_ (–9.5 ± 1.6‰; **Figure 7A**) varied with depth (*p* < 0.05), with higher δ^18^O_soil_ (–8.1 ± 1.4‰) in top soil layer (0–5 cm depth) and lower δ^18^O_soil_ (–10.2 ± 1.2‰) in subsoil layers (5–20 cm depth). δ^18^O_root_ was within the range of δ^18^O_soil_ (**Figures 7A, B**), indicating soil water as the main source of plant water. δ^18^O_root_ of *Alchemilla* and *Trifolium* was between the topsoil and the subsoil δ^18^O_soil_, whilst δ^18^O_root_ of *Poa* and *Taraxacum* was close to subsoil δ^18^O_soil_. δ^18^O_root_ varied with plant genera (*p* < 0.05; **Figure 7B**), with higher δ^18^O_root_ for *Alchemilla* and *Trifolium* (–9.0 ± 1.0‰), but lower δ^18^O_root_ for *Poa* and *Taraxacum* (–10.6 ± 1.0‰). The comparison of δ^18^O_soil_ and δ^18^O_root_ (**Figure 7B**) indicated that *Alchemilla* and *Trifolium* used shallower soil water as compared to *Poa* and *Taraxacum*.

Across all four genera, leaf water δ^18^O_lw_ (–2.6 ± 4.8‰; **Figure 7C**) was on average higher than δ^18^O_root_ (–9.8 ± 1.3‰; **Figure 7B**), indicating evaporative processes of leaf water as compared to xylem water. *Poa* had the highest δ^18^O_lw_ among the four genera (*p* < 0.05; **Figure 7C**), indicating strongest water stress of *Poa* derived from their stronger evaporation or stress-induced partial stomatal closure. *Taraxacum* tended to have the lowest δ^18^O_lw_, but not significantly different from *Alchemilla* and *Trifolium* (*p* ≥ 0.05). δ^18^O_dew_ changed over time, with –7.5 ± 0.4‰ at 03:00 of IOP1, increasing to –6.7 ± 1.0‰ at 00:00 and –5.7 ± 0.5‰ at 03:00 during IOP2 (*p* ≥ 0.05; **Figure 7B**). δ^18^O_eq_ of the liquid water in equilibrium with atmospheric water vapor was –5.0‰ to –4.7‰ during 20:30 to 03:00 of IOP2. Compared to the local meteoric water line (LMWL; δ^2^H = 7.83 δ^18^O + 12.97; following Prechsl et al., 2014), δ^2^H_eq_–δ^18^O_eq_ was above the LMWL. In contrast, all δ^2^H–δ^18^O pairs for dew droplets, plants and soil water fell below the LMWL, indicating evaporation of these water pools compared to local precipitation, particularly of leaf water (**Figure 7D**).

#### 3.3.3 Effect of isotopically labelled dew on leaf water and sugar isotopes

**Figure 8:**
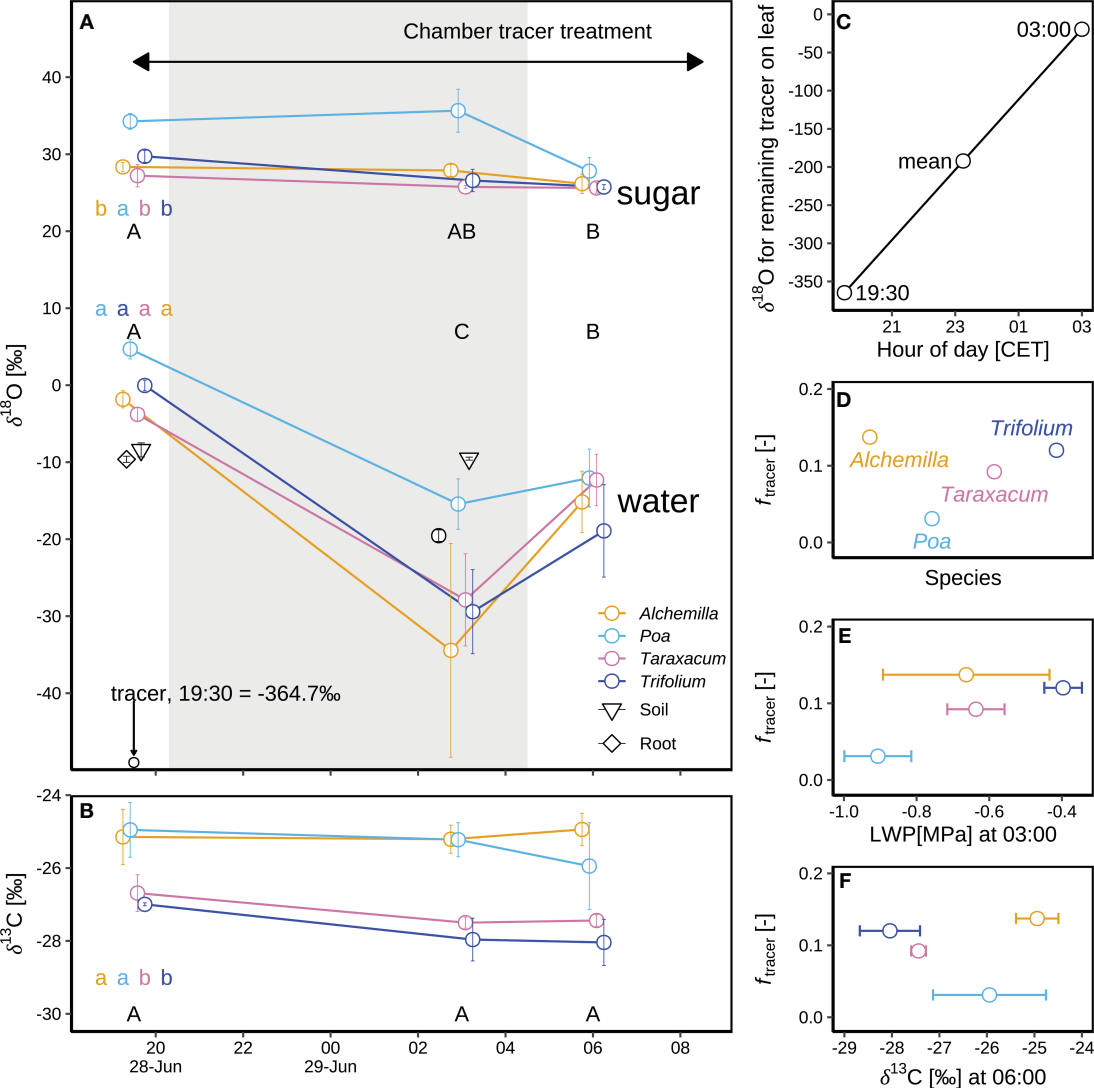
Oxygen isotope composition of leaf sugars (δ^18^O_ls_ and δ^13^C_ls_) and leaf water (δ^18^O_lw_) in a chamber tracer experiment during an intensive observation period (IOP1) for four plant genera (*Alchemilla*, *Poa*, *Taraxacum*, and *Trifolium*) at the end of the June 2019 heatwave: **(A)** Mean and standard errors of mean (SEM) for the isotope composition of bulk leaf sugar (δ^18^O_ls_) and leaf water (δ^18^O_lw_) with the corresponding xylem water of root crown, soil water (at 5 cm soil depth) and tracer on leaf surface. The grey shaded area corresponds to the nocturnal period. Leaf samples taken before sunset in IOP1 (19:30 CET) acted as a control for natural isotope abundances. **(B)** Mean and SEM for the carbon isotope composition of bulk leaf sugar (δ^13^C_ls_) for four genera. **(C)** Changes in δ^18^O of tracer remaining on the leaves, from 19:30 (δ^18^O_tracer_19:30_) when the tracer was sprayed on the leaves until 03:00 (δ^18^O_tracer_03:00_) before sunrise; the mean tracer δ^18^O (δ^18^O_tracer_mean_) was calculated as arithmetic mean. **(D)** Contributions of δ^18^O_tracer_ in leaf water (*f*_tracer_) at 03:00 on 29 June 2019 are based on a two-pool mixing model. **(E)** Correlation of *f*_tracer_ with leaf water potential (LWP) for four genera. **(F)** Correlation of *f*_tracer_ with δ^13^C_ls_. Different letters in panels a–b indicate statistically significant difference (*p* < 0.05) of leaf water and sugar with capital letters representing temporal differences and lower-cases representing among-genera differences over IOP1.

Adding isotopically ^18^O-depleted water as a tracer in the chamber tracer experiment on IOP1 night induced a substantial ^18^O-depletion in leaf water (δ^18^O_lw_; *p* < 0.05), but not so in leaf sugars (δ^18^O_ls_; *p* ≥ 0.05; **Figure 8A**). Before applying the tracer at 19:30 (before sunset), δ^18^O of leaf sugar (δ^18^O_ls_) for the four genera (29.9 ± 2.9‰; **Figure 8A**) was 30.1‰ higher than their respective δ^18^O_lw_ (–0.2 ± 3.4‰). During the following 7.5 h overnight (until 03:00), δ^18^O_lw_ of the four genera decreased by a further 26.6 ± 10.4‰ as compared to before-sunset levels. In contrast, δ^18^O_ls_ (29.2 ± 4.5‰) of the four genera did not change after tracer amendment, and consequently δ^18^O_ls_ of the four genera was 55.7‰ higher than the respective δ^18^O_lw_ (**Figure 8A**). Wet foliage due to tracer application persisted for 10.5 h, thus after sunrise (06:00), δ^18^O_lw_ of the four genera increased to –14.6 ± 4.7‰ in respect to predawn δ^18^O_lw_, whereas the corresponding δ^18^O_ls_ (26.3 ± 1.3‰) remained almost constant over the night until sunrise, indicating minor effect of amended tracer on soil moisture. As a result, δ^18^O_ls_ of the four genera after sunrise was 40.9‰ higher than the corresponding δ^18^O_lw_ (**Figure 8A**).

The changes in δ^18^O_lw_ varied by genus before and during the chamber tracer experiment. Before sunset and tracer amendment, *Poa* δ^18^O_lw_ (4.7 ± 1.3‰) was 6.6‰ higher than δ^18^O_lw_ of the other three genera (–1.9 ± 1.8‰; **Figure 8A**), indicating more severe water stress of *Poa*. However, the difference for predawn (03:00) δ^18^O_lw_ of *Poa* (–15.4 ± 2.8‰; **Figure 8A**) increased to 15.3‰ compared to the corresponding δ^18^O_lw_ of the other three genera (–30.7 ± 8.9‰; **Figure 8**), which might be due to the stronger evaporation and less foliar water uptake of *Poa*, or stress-induced partial stomatal closure. Overall, the ranking of the genera stayed relatively stable, with *Poa* typically showing the highest δ^18^O_lw_.

During the experiment, δ^18^O of the added tracer increased from –364.7‰ at 19:30 to –19.5 ± 1.8‰ at 03:00 (**Figure 8C**). The contribution (*f*_tracer_) of the added tracer (δ^18^O_tracer_mean_ of –192.1‰) to plant leaf water was 3–14%, highest for *Alchemilla* and lowest for *Poa* (**Figure 8D**). *f*_tracer_ was positively correlated with the corresponding predawn LWP, except for *Alchemilla* with large variability in their predawn LWP (**Figure 8E**).

δ^13^C_ls_ was rather constant over the IOP1 night. Higher δ^13^C_ls_ of *Alchemilla* and *Poa* indicated their lower WUE, as compared to *Taraxacum* and *Trifolium* (**Figure 8B**). δ^13^C_ls_ of *Poa*, *Taraxacum* and *Trifolium* was negatively correlated with *f*_tracer_ (**Figure 8F**), whilst *Alchemilla* with highest *f*_tracer_ showed highest δ^13^C_ls_ and thus lowest WUE.

### 3.4 Controls of atmospheric and soil conditions on plant water

**Figure 9:**
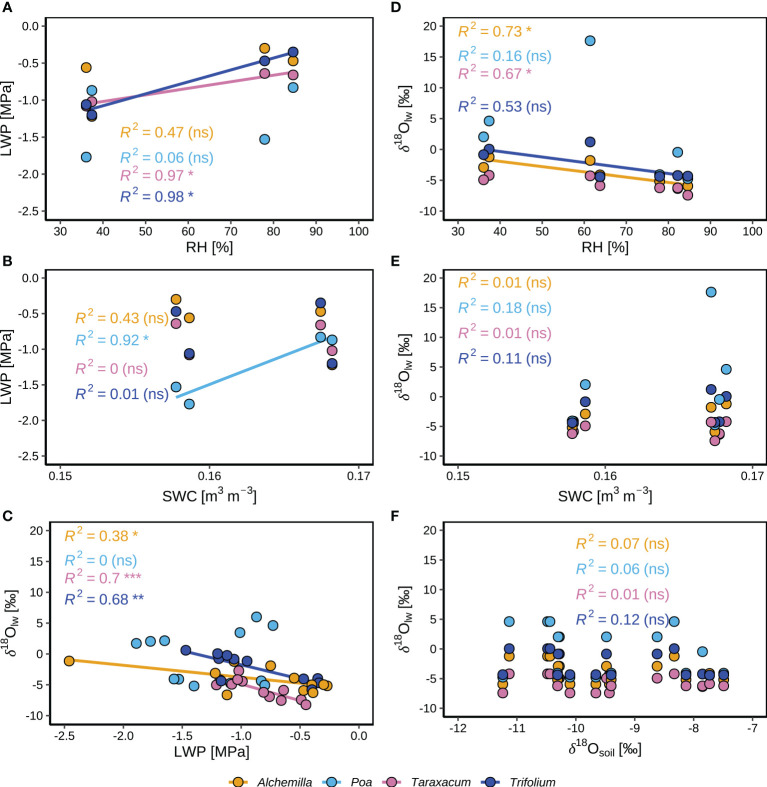
Correlations between different environmental and plant variables for four plant genera (*Alchemilla*, *Poa*, *Taraxacum*, and *Trifolium*) during two consecutive intensive observation periods (IOP1 and IOP2) with nocturnal dew formation at the end of the June 2019 heatwave: **(A)** Median leaf water potential (LWP) and corresponding relative humidity (RH, at 2 m agl). **(B)** Median LWP and corresponding volumetric soil water content (SWC) at 5 cm depth. **(C)** Leaf water isotopes (δ^18^O_lw_) and LWP. **(D)** Meidian δ^18^O_lw_ and corresponding RH. **(E)** Median δ^18^O_lw_ and corresponding SWC. **(F)** Median δ^18^O_lw_ and median soil water isotopes (δ^18^O_soil_).

LWP of *Taraxacum* and *Trifolium* were positively correlated with RH (**Figure 9A**), indicating the controls of atmospheric humidity on their LWP. LWP of *Poa* was positively correlated with SWC (**Figure 9B**), indicating the controls of soil moisture on their LWP. This corresponded to the significant decline of *Poa* LWP at the end of the heatwave (**Figure 6**), under the conditions of low SWC (0.15 m^3^ m^-3^; **Figure 2C**) close to wilting point. LWP and δ^18^O_lw_ of *Alchemilla*, *Taraxacum* and *Trifolium* were negatively correlated (**Figure 9C**), indicating the controls of leaf water content on their δ^18^O_lw_. Stronger drought-stress of *Poa* might induce partial stomatal closure, and thus result in their more enriched leaf water isotopes (higher δ^18^O_lw_) not relevant to LWP. The slightly improved *Alchemilla* LWP at the end of the heatwave might be derived from the accumulated benefits by dew, which contributed more to *Alchemilla* leaf water (14%; **Figure 8D**) compared to the other three genera (≤ 12%). δ^18^O_lw_ of *Alchemilla*, and *Taraxacum* was negatively correlated with RH (**Figure 9D**), indicating the control of atmospheric conditions on their δ^18^O_lw_. δ^18^O_lw_ did not show significant correlation with SWC and δ^18^O_soil_ (**Figures 9E, F**), indicating the minor control of soil water on leaf water isotopes.

## 4 Discussion

### 4.1 Dew benefits cancelled out by heat-drought stress

**Figure 10:**
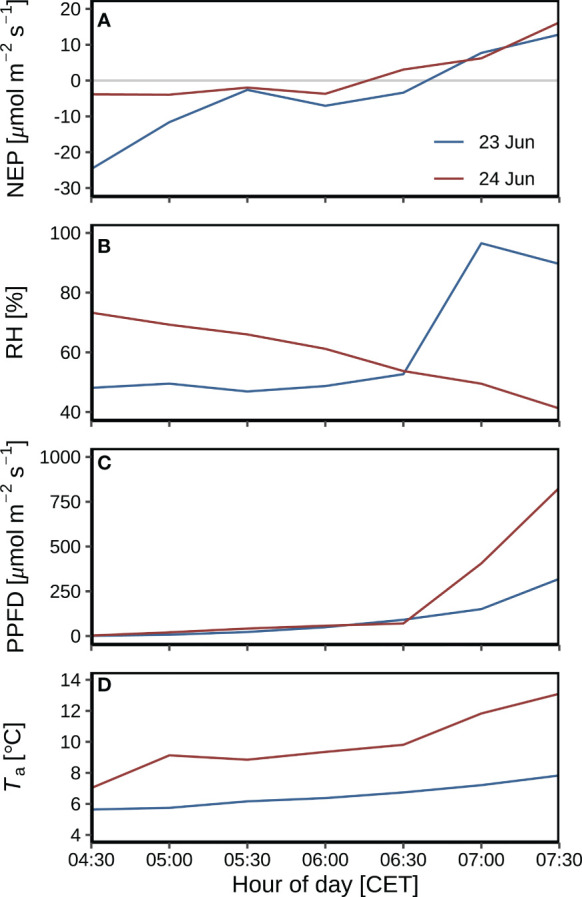
Environmental variables during leaf wetting by dew in the early mornings of 23-24 June before the heatwave: **(A)** Net ecosystem production (NEP). **(B)** Leaf wetness. **(C)** Photosynthetic photon flux density (PPFD). **(D)** air temperature (*T*_a_) at 2 m agl.

The benefits of dew on NEP were observed in the early morning hours of 23–24 June before the heatwave (**Figure 10**). From sunrise to 06:30 on 23 and 24 June, PPFD was at similar levels (**Figure 10C**), hence higher NEP on 24 June (**Figure 10A**) might be induced by higher temperature (**Figure 10D**) as compared to 23 June. But from 06:30 to 07:30, although higher PPFD and temperature on 24 June, NEP was at the similar levels as that on 23 June (**Figure 10C**), probably induced by higher potential of dew formation as indicated in higher RH on 23 June (**Figure 10B**).

However, with the occurrence of heatwave, dew benefits on NEP were cancelled out, as shown in the reduced NEP with increasing RH (**Figure 5C**). The chamber tracer experiment at the end of the heatwave showed that dew isotope signal was not transferred to leaf sugar (**Figure 8A**), indicating that dew water did not participate in carbon assimilation during the heatwave. The possible reason could be derived from the minor contribution (3–14%) of dew water to plant leaf water (**Figure 8D**), corresponding to previous research that foliar water uptake can only increase leaf water content by 2–11% (Limm et al., 2009). Due to the heat-drought stress, partial stomatal closure and vapor pressure gradient (Li et al., 2023) from leaf to atmosphere (saturated leaf internal environment vs unsaturated atmospheric conditions) might limit the uptake of dew water *via* leaf, thus most of the dew water during the heatwave could evaporate after sunrise instead of being used for carbon assimilation. Oliveira et al. (2021) showed that dew evaporation processes induced CO_2_ loss of a maritime pine forest during the rain-free period after wildfire, but Simonin et al. (2009) reported that the reduction in leaf water deficit by fog water can result in improved carbon gain. Therefore, the effect of dew on ecosystem exchange varied by environmental conditions, e.g., environmental stress (Oliveira et al., 2021), the duration of canopy wetting (Simonin et al., 2009), the wettability of the leaf surface (Brewer and Smith, 1994; Hanba et al., 2004), and the foliar water uptake capacity of plants (Simonin et al., 2009).

Our tracer chamber experiment was carried out in a single chamber, and with only once sampling after sunrise, thus it was not possible to investigate the effect of dew on carbon assimilation after dew totally evaporating from surfaces. Future research on hourly resolution and longer period of after-sunrise isotope measurements is recommended to answer this question.

### 4.2 Ecosystem water and carbon exchange

Despite heat-drought stress, alpine grassland kept high ET during the heatwave (**Figure 3A**), as long as soil moisture was available (**Figure 2C**) to meet its evaporative demand (Teuling et al., 2010; Wolf et al., 2013).

De Boeck et al. (2016) showed that a combined heatwave and drought stress induced a reduction in above-ground biomass of alpine grassland plants. In this study, the reduction of NEP by the heat-drought stress was most pronounced during the two hottest days (26 and 27 June; **Figures 4D, E**) of the heatwave period, Compared to 26 June (25.4 °C; **Figure 4B**), 27 June showed a longer period of net carbon emission (**Figure 4E**) despite lower maximum *T*_a_ (24.8 °C; **Figure 5E**) and SWC (**Figure 2C**). This might be explained by an intensifying effect of drought stress, corresponding to previous research by De Boeck et al. (2016), and the influence of severe heat stress on nighttime refilling. Nevertheless, heat stress was a bit relieved after the heatwave peak, when temperatures only reached 21.5 °C, and NEP recovered immediately. The rapid re-growth ability of alpine plants after heat-drought stress (Brilli et al., 2011) might explain the recovery of NEP during 28–30 June (**Figure 3B**) after the hottest two days. This also corresponded to the fact that LWP of plants (except *Poa*) did not significantly decreased at the end of the heatwave (**Figure 6**). The refilling of plant tissues during the night is a well-known phenomenon (Schulze et al., 2019), helping to relieve water losses during heat-drought stress, and might contribute to the recovery of diurnal NEP on 28–30 June after the peak of the heatwave to the levels slightly lower than the beginning of the heatwave on 23–25 June. The redistribution of soil water by deeper plant roots to the shallower soil depths might provide sufficient soil moisture sources for plant tissue refilling (Burgess and Bleby, 2006) after the peak of the heatwave. This may also explain why the predawn LWP of *Alchemilla*, *Taraxacum*, and *Trifolium* did not decline at the end of the heatwave. Yet, the nighttime refilling mechanism of plant tissues might not always lead to a full recovery of NEP at daily scales, particularly in the case of extreme heat-drought stress. As a result, the most severe decline of NEP was observed during the peak of the heatwave on 26–27 June. The most severe heat stress on 26 June might cause the suppression of tissue refilling during the following night, and thus more pronounced NEP reduction was observed on the next day (27 June) instead of on the hottest day (26 June). The decline of *Poa* LWP (**Figure 6**) might be due to their more severe drought stress (as indicated in their higher δ^18^O_lw_ as compared to the other three genera; **Figures 7A**, **8A**) and dependence on soil moisture (**Figure 9B**). The decline of Poa LWP had minor effect on NEP, probably due to their small plant size and stress-induced partial stomatal closure. We could not quantify the individual contribution of different genera on ecosystem water and carbon fluxes, and effect of eddy-covariance footprint; thus, we suggest that future research can combine ecosystem-scale eddy-covariance fluxes, footprint models and plant-scale chamber fluxes, complemented by plant community compositions to quantify the effect of different genera on ecosystem exchange.

Dew amount was found to be underestimated by eddy-covariance H_2_O fluxes because dew occurs on clear and calm nights with stably stratified nocturnal boundary layer (Jacobs et al., 2006; Li et al., 2021). High accuracy weighing lysimeters can be an option to quantify dew amount into ecosystems (Riedl et al., 2022; Ucles et al., 2013). CO_2_ fluxes can be measured by eddy-variance (Eugster and Siegrist, 2000) and laser (Maier et al., 2022) approaches. For alpine ecosystems, a challenge is the topographic variability that induces large uncertainties of CO_2_ fluxes (Hammerle et al., 2007). Due to the low quality of CO_2_ fluxes during nighttime by eddy-covariance measurements, we could not assess the dew effect in darkness. Furthermore, the benefits of dew on ecosystems can continue after dew drying out on vegetation surfaces, which could be traced by high-resolution measurements of H_2_O and CO_2_ isotopes, but was not possible in this study based on 3–13 h intervals of destructive isotope sampling. Therefore, additional methods, e.g., synchronized and continuous laser measurements of H_2_O and CO_2_ fluxes (Li et al., 2021; Maier et al., 2022) and their isotopic fluxes (Siegwolf et al., 2021) at both plant and ecosystem scales need to be explored to assess the long-term benefits of dew at plant (e.g., among species difference) and ecosystem scales.

### 4.3 Genus variability

Plant water stress can be induced by low soil moisture or high atmospheric water demand (Liu et al., 2020). In this study, leaf water status of *Poa* was dependent on soil moisture (**Figure 9B**), whilst LWP of *Alchemilla*, *Taraxacum*, and *Trifolium* were mainly controlled by atmospheric humidity conditions (**Figure 9C**). Among the four genera, *Poa* was most substantially affected by heat-drought stress as indicated in their lower LWP (**Figure 6**), higher δ^18^O_lw_ and δ^13^C_ls_ (**Figures 7A**, **8A, B**). This could be induced by the reliance of *Poa* on soil moisture (**Figure 9B**), and lower foliar water uptake of *Poa* (**Figure 8D**). More severe drought stress of *Poa* induced their lower WUE (higher δ^13^C_ls_; **Figure 8F**) as compared to *Taraxacum*, and *Trifolium*. Palmately-lobed and hairy leaves of *Alchemilla* might prolong the dew water retention on their leaves, and thus induced stronger foliar water uptake (**Figure 8D**) and slightly increased LWP (**Figure 6**) compared to the other three genera.

Both *Alchemilla* and *Trifolium* depended on shallower soil water depth (**Figure 7B**), but they probably benefited from dew water to maintain their plant water status (**Figure 6**) in response to heat and drought stress. In the case of *Trifolium*, the hairy trichomes on the edges of the leaves probably promoted foliar water uptake (**Figure 8D**). Many high-elevation plants have hairy structures to help reduce water loss, reflect excess radiation, and protect plants from pathogens (Zeng et al., 2013; Hamaoka et al., 2017). The control experiment at the same site by Prechsl et al. (2015) found that C_3_-grasses did not shift to deeper soil water under drought treatment, indicating that high-elevation plants could benefit from leaf structures regulating their energy and water balances. On the contrary, with deeper soil water sources, the plant water status of *Poa* strongly declined (**Figure 6**) in response to heat-drought stress. The maintenance of *Taraxacum* LWP (**Figure 6**) in response to heat-drought stress might be beneficial from their waxy leaf surfaces (**Figure S1**) and deeper soil water uptake (**Figure 7B**). These results indicated that both soil moisture and atmospheric conditions can affect the ecosystem carbon and water exchange under heat-drought stressed conditions through their varied influence on different plant genera. Due to the similar δ^18^O values for dew and soil water (**Figure 7B**), we could not split the contributions of dew (foliar water uptake) and nighttime plant tissue refilling (root water uptake; Schulze et al., 2019) on plant water status using the natural-conditioned data, because both root water uptake and foliar water uptake could occur during nighttime, and dew water can also drip off to the soil (Dawson, 1998) and disturb the isotopic signal of root water uptake fluxes. But our chamber-tracer experiment indicated that the contribution of dew on leaf water can vary from 3% to 14% (**Figure 8D**). We did not isolate the soil from the vegetation when amending tracer on the grassland plots, hence the tracer could have been directly applied on the soil, and the tracer sprayed on leaf surfaces could also have dripped into the soil. But according to the slight depletion of δ^18^O_lw_ in our chamber-tracer experiment, the drip-off effect of dew was probably minor compared to direct foliar uptake of dew and atmospheric water vapor. Based on the facts of similar isotopic signal of dew and soil moisture in natural conditions, previous research used excised leaves and isotopically depleted/enriched water to distinguish the two (root and foliar) water sources (Kim and Lee, 2011; Goldsmith et al., 2017). However, these controlled experiments were performed with self-made chambers acting as a heat trap preventing radiative cooling, which is the most important driver of dew formation in natural conditions (Curtis, 1936; Li et al., 2023), and may thus not reflect natural conditions. Future research should therefore apply approaches that allow to estimate the dew influence on plant water under varying soil moisture conditions in the field (Li et al., 2021).

## 5 Conclusions

The combination of stable isotope analyses in meteoric waters and leaf sugars, meteorological and plant physiological measurements, complemented by eddy-covariance fluxes for H_2_O vapor and CO_2_ provided novel insights into the effects of combined heat-drought stress on the water and carbon exchange of an alpine grassland.

(1) Before the heatwave, NEP increased with RH levels, but the dew benefits were cancelled out during the heatwave. NEP decreased with RH levels at the beginning of the heatwave, and showed no significant correlation with leaf wetness at the later stages of the heatwave.
(2) The isotope signal of amended dew in the chamber tracer experiment was not transferred to leaf sugar, indicating that dew water did not participate in the carbon assimilation. The minor effect of dew on NEP might be derived from low contribution (3-14%) of dew in leaf water, and the partial stomatal closure induced by heat-drought stress.
(3) NEP reduction was most severe on the hottest two days, with the shift from net ecosystem uptake to net ecosystem emission on the second hottest day just after the peak of the heatwave, indicating that the heat effect was intensified by drought stress.
(4) The recovery of NEP after the peak of the heatwave indicated the regrowth ability of alpine plants. Plants benefited from the minor effect of heat-drought stress on their water status, which could be recovered *via* the refilling of plant tissues during nighttime.
(5) The among-genera difference of leaf water status and isotopes in response to heat-drought stress and dew occurrence indicated the varied controls of soil moisture and atmospheric evaporative demand on plant water status, with soil-dependent genera suffering from more severe drought stress compared to the atmospheric-reliant genera.

Our results thus reveal that dew influence on ecosystem water and carbon exchange varied by the levels and stages of environmental stress and plant physiology.

## Data availability statement

The datasets presented in this study can be found in the ETH Zurich research collection at https://doi.org/10.3929/ethz-b-000537314.

## Author contributions

YL, WE, AR, and ML designed the experiment. YL conducted the field and laboratory work. YL and ML performed the purification of plant sugars. YL wrote and revised the manuscript, with contributions and feedback from NB, WE, ML, FA and AR. All authors contributed to the article and approved the submitted version. WE passed away on 23 May 2022 before the final submission of this study.
