# Supplementary material for: Dew benefits on alpine grasslands are cancelled out by combined heatwave and drought stress
Source: Front Plant Sci. 2023 May 9;14:1136037. doi: 10.3389/fpls.2023.1136037 (PMC10203623; doi:10.3389/fpls.2023.1136037)
Supplement: Supplementary file 1 [file DataSheet_1.docx]

Supportive information

Dew benefits on alpine grasslands are cancelled out by combined heatwave and drought stresses

**Yafei Li^1*^, Werner Eugster^1^, Andreas Riedl^1^, Marco M. Lehmann^2^, Franziska Aemisegger^3^, Nina Buchmann^1^**

^1^Institute of Agricultural Sciences, ETH Zurich, Zurich, Switzerland;

^2^Forest Dynamics, Swiss Federal Institute for Forest, Snow and Landscape Research (WSL), Birmensdorf, Switzerland

^3^Institute for Atmospheric and Climate Science, ETH Zurich, Zurich, Switzerland

*** Correspondence:**

Corresponding Author

Yafei Li

l.yafei@outlook.com; yafei.li@usys.ethz.ch


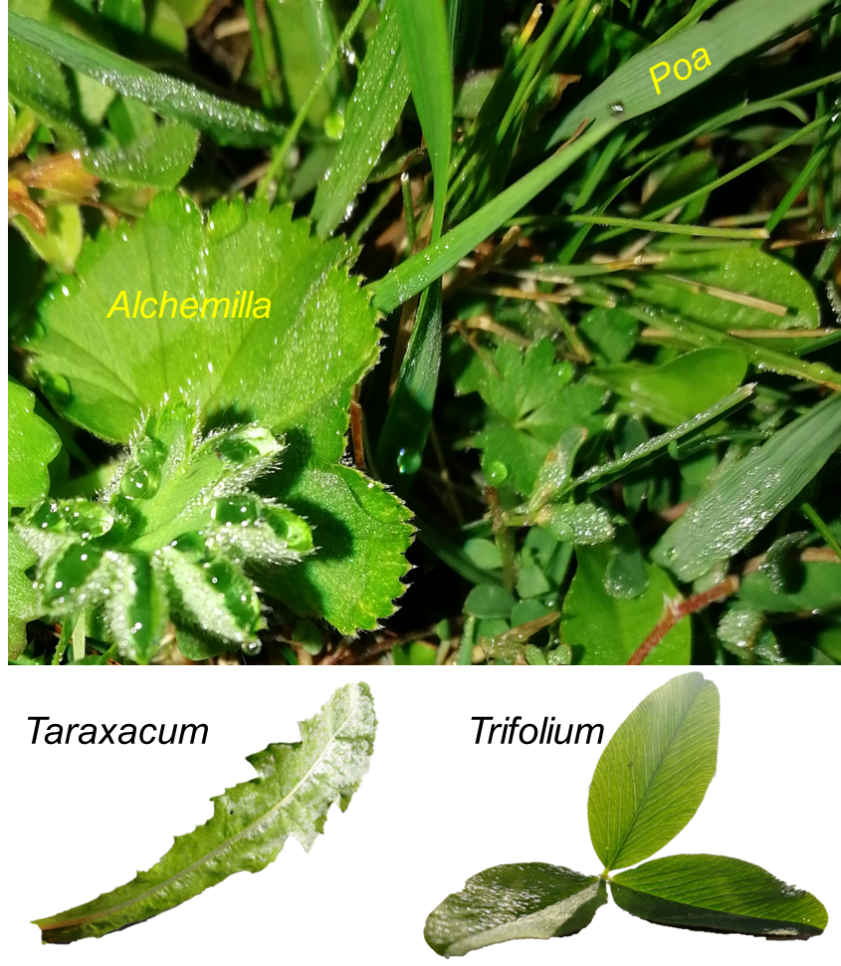


**Fig. S1** Images of *Alchemilla*, *Poa*, *Taraxacum*, and *Trifolium* leaves.

**Fig. S2** Comparison of leaf wetness measured by BNS sensor (G. Lufft Mess-und Regeltechnik GmbH, Fellbach, Germany) and a more accurate leaf wetness sensor (PHYTOS 31, Meter Group AG, Munich, Germany) at a later time (5–6 July 2020) of our observation campaigns. The BNS sensors overestimated the leaf wetting duration, and the termination of leaf wetting was defined as the point when leaf wetness by BNS steeply and linearly decreased.
